# Alternative Nuclear Imaging Tools for Infection Imaging

**DOI:** 10.1007/s11886-022-01708-2

**Published:** 2022-06-13

**Authors:** Paola Anna Erba, Francesco Bartoli, Martina Sollini, Berchiolli Raffaella, Roberta Zanca, Esposito Enrica, Elena Lazzeri

**Affiliations:** 1 Regional Center of Nuclear Medicine, Department of Translational Research and Advanced Technologies in Medicine and Surgery, University of Pisa, Via Roma 57, 56126 Pisa, Italy; 2 Medical Imaging Center, University of Groningen, University Medical Center Groningen, Groningen, The Netherlands; 3 Department of Biomedical Sciences, Humanitas University, Pieve Emanuele, Italy; 4 IRCCS Humanitas Research Hospital, Rozzano, Italy; 5 Vascular Surgery Unit, Department of Translational Research and Advanced Technologies in Medicine and Surgery, University of Pisa, Pisa, Italy

**Keywords:** CVS infections, Infective endocarditis, CIED infections, WBC scan, SPECT/CT embolic burden

## Abstract

**Purpose of Review:**

Cardiovascular infections are serious disease associated with high morbidity and mortality. Their diagnosis is challenging, requiring a proper management for a prompt recognition of the clinical manifestations, and a multidisciplinary approach involving cardiologists, cardiothoracic surgeons, infectious diseases specialist, imagers, and microbiologists. Imaging plays a central role in the diagnostic workout, including molecular imaging techniques. In this setting, two different strategies might be used to image infections: the first is based on the use of agents targeting the microorganism responsible for the infection. Alternatively, we can target the components of the pathophysiological changes of the inflammatory process and/or the host response to the infectious pathogen can be considered. Understanding the strength and limitations of each strategy is crucial to select the most appropriate imaging tool.

**Recent Findings:**

Currently, multislice computed tomography (MSCT) and nuclear imaging (^18^F-fluorodeoxyglucose positron emission tomography/computed tomography, and leucocyte scintigraphy) are part of the diagnostic strategies. The main role of nuclear medicine imaging (PET/CT and SPECT/CT) is the confirmation of valve/CIED involvement and/or associated perivalvular infection and the detection of distant septic embolism. Proper patients’ preparation, imaging acquisition, and reconstruction as well as imaging reading are crucial to maximize the diagnostic information.

**Summary:**

In this manuscript, we described the use of molecular imaging techniques, in particular WBC imaging, in patients with infective endocarditis, cardiovascular implantable electronic device infections, and infections of composite aortic graft, underlying the strength and limitations of such approached as compared to the other imaging modalities.

## Introduction

Infective endocarditis (IE) is a complex and deadly disease with an incidence of IE varying from one country to another, within a range of 3–10 episodes/100000 people per year [1]. Infections of prosthetic valve and electronic implantable cardiac device (CIED) represent the majority of healthcare-associated infection.

Prosthetic valve IE (PVIE), cardiovascular implantable electronic device IE (CDRIE), nosocomial, staphylococcal, and enterococcal IE are currently the more frequent IE variants [2•]. IE is associated with an unacceptably poor prognosis with a mortality of about 17.1% as recently showed by data from the EURO-ENDO registry [2•] as a consequence of the increasing proportion of older patients with more severe disease, changing epidemiological profiles, and greater numbers of patients with prosthetic valve or device-related infection [1, 3]. Interestingly, mortality was particularly high in EURO-ENDO, when surgery was indicated but not performed, emphasizing the role of an aggressive surgical strategy in these patients.

Infection is also one of the most serious complications of CIED implantation being associated with significant morbidity, mortality, and healthcare costs [4]. Staphylococcal species, both *Staphylococcus aureus* and coagulase-negative staphylococci, account for about 60–70% of CIED infections [5]. In-hospital mortality is estimated around 5–10% [6–8] while 1-year all-cause mortality ranges between 16 and 36% [9, 10] although both appear to be reducing over time [10, 11].

A particular rare case of IE is the one arising after the corrections of aorta defects with the Bentall procedure [12]. It is reported in about 3% of the cases. *Staphylococcus aureus* is the predominant cause (35%) of infection after Bentall procedure with a recent 20% increase in methicillin-resistant *Staphylococcus aureus* infections [13] associated with VPI (Vascular Prosthetic Infection) [14]. Despite their low incidence, such infections are severe and hard to treat, with high mortality associated with the replacement of the graft, especially in cases of long-lasting infections or severe co-morbidities.

Overall, IE and CIED infections are challenging and their management requires a prompt recognition of the clinical manifestations, which can vary significantly, and a multidisciplinary approach involving cardiologists, cardiothoracic surgeons, infectious diseases specialist, imagers, and microbiologists.

Microbiological tests for germ characterization along with imaging (mainly echocardiography) are necessary for the diagnosis of cardiovascular infection, according to established criteria such as the modified Duke criteria [15, 16•]. However, a number of patients are misclassified as “possible” IE in about 25% of the cases of pathologically proven endocarditis [16•] having negative blood culture [17–19] or negative/inconclusive echocardiography. Therefore, over time refinements of the diagnostic criteria have been implemented to improve diagnosis—and consequently prognosis—of IE and CIED infections. The ESC Guidelines on the management of IE were published in 2015 which integrated other non-invasive imaging techniques in the diagnostic algorithm of IE [20••]. Accordingly, although echocardiography remains the pillar in the diagnostic workflow of IE, multislice computed tomography (MSCT) and nuclear imaging (^18^F-fluorodeoxyglucose positron emission tomography/computed tomography, and leucocyte scintigraphy) [21••, 22, 23••, 24••, 25•] were introduced in clinical practice, after being proved to positively impact on early and accurate diagnosis [2•]. More recently, also the American Heart Association (AHA)/American College of Cardiology (ACC) recommended PET/CT in the management of patients with IE (class 2a) as part of the 2020 Guideline for the Management of Patients with Valvular Heart Disease [26]. Similarly, the crucial role of imaging has been also recognized for pocket or CIED infections. Indeed, the Novel 2019 International CIED infection criteria have moved to include both clinical data and imaging findings in patient workout [27••].

**Fig. 1:**
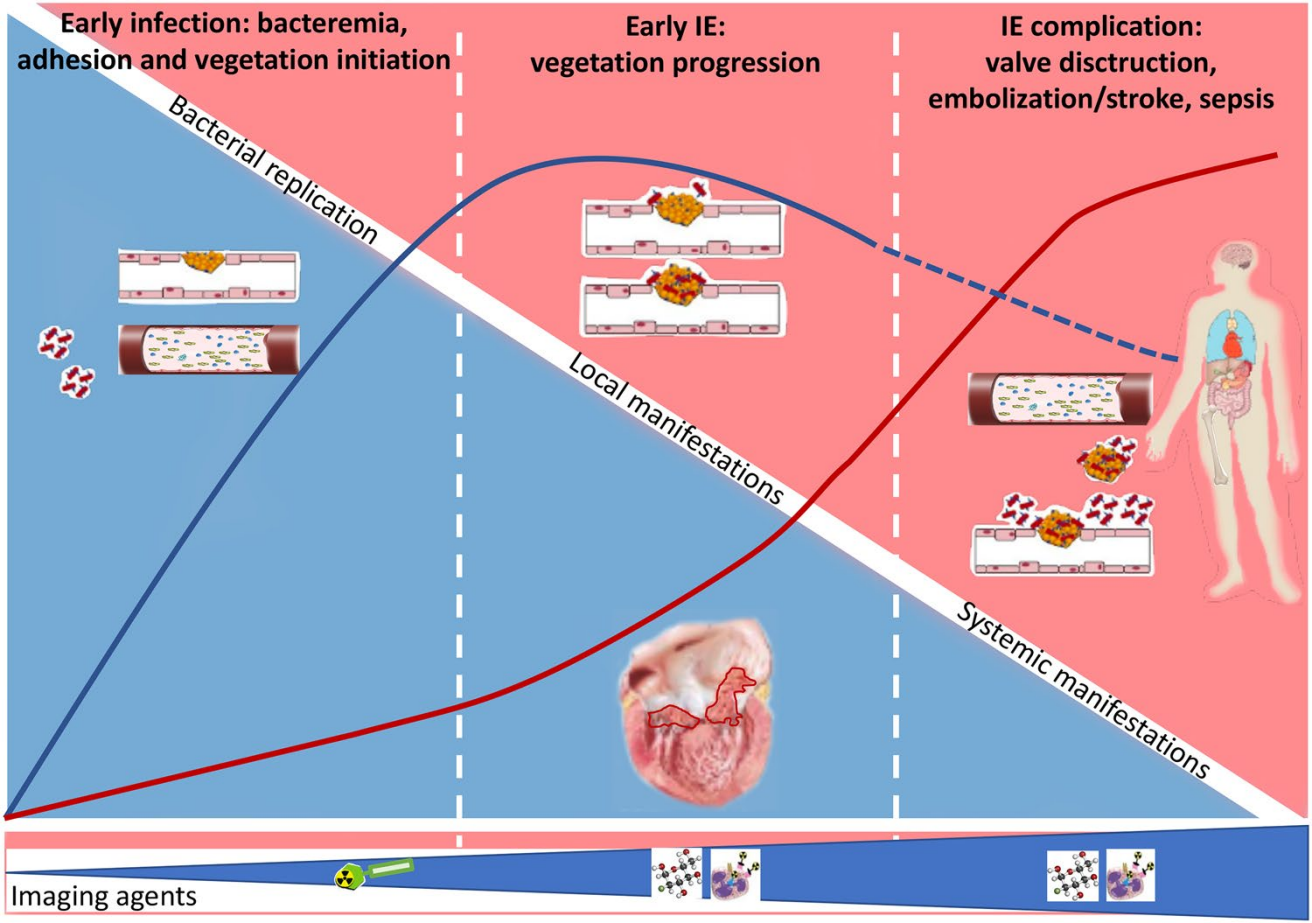
Schematic representation of the IE pathogenesis from the microorganism entrance and subsequent heart native valve/prosthetic valve adhesion to local and systemic manifestation of the disease. In the **lower panel**, the type of radiopharmaceutical agents to be used in relation to the different disease phase: bacterial specific agents potentially leading to early diagnosis or agents identifying the host immune response to infections such as WBC imaging and [^18^F]FDG. The *blue curve* indicates the intensity of the local infection burden while the *red curve* the intensity of systemic infection

The main role of nuclear medicine imaging (PET/CT and SPECT/CT) is the confirmation of valve/CIED involvement and/or associated perivalvular infection and the detection of distant septic embolism (Fig. 1).

Two different strategies might be used to image infections: the first is based on the use of agents targeting the microorganism responsible for the infection. Alternatively, we can target the components of the pathophysiological changes of the inflammatory process and/or the host response to the infectious pathogen can be considered.

Biochemical differences in metabolism, proteins, and cell wall components between bacteria and mammalian cells provide opportunities for developing bacteria-specific imaging agents. They consist of antibiotics, antimicrobial peptides, or small molecules selectively metabolized by pathways expressed only in bacteria [28] (Table 1). Although promising, many radiolabeled antibiotics or antimicrobial peptides have failed in reliably differentiating infection from sterile inflammatory processes [29–31]. The most recent discovery in this field is a PET agent based on the broad-spectrum antibiotic trimethoprim (TMP), [^18^F]FPTMP, which has over 30,000-fold selectivity for bacterial target over the human homolog. Encouraging results have been demonstrated in animal models [32].

**Table 1:** Summary of the main antibiotics, antimicrobial peptides, or small molecules selectively metabolized by pathways expressed only in bacteria

Class	Examples of radiolabeled molecules	References
Bacterial cell wall synthesis	d-Amino acids analogues ^99m^Tc-fluoroquinolones ^99m^Tc-cephalosporins	[38]
Bacterial folate pathway	[^11^C]-*para*-aminobenzoic acid (PABA) and 2-[^18^F]-PABA	[33, 35]
Bacteriophage	^99m^Tc-labeled bacteriophages	[68]
Cytoplasmic membrane	^123^I-amphotericin-B ^99m^Tc-UBI 29–41, ^68^ Ga-NOTA-UBI 29–41	[69, 70]
DNA gyrase	^99m^Tc/^18^F-ciprofloxacin	
DNA synthesis	[^18^F]-trimethoprim [^18^F]FPTMP	
Gram-negative Enterobacteriaceae	2-[^18^F]-fluorodeoxysorbitol ([^18^F]-FDS) ^18^F-fluoromaltohexaose (FMH) ^18^F-fluoromaltotriose ^18^F-fluoroacetamido-d-glucopyranose (FAG) ^99m^Tc-polymyxin B	[40]
Iron metabolism	Siderophore-derived agents	[71]
Maltodextrin transporter	[^18^F]-labeled maltohexaose and 6-[^18^F]-fluoromaltotriose	[36, 37]
Nucleoside analogues	^124^I-Fialuridine (FIAU)	
Protein synthesis (16S, 30, and 50S inhibitors)	^99m^Tc-erythromicycin ^99m^Tc-MORF ^99m^Tc-kanamycin	
Vitamins	Biotin	

An alternative approach is the development bacteria-specific radiolabeled molecular imaging agents based on prokaryotic metabolism [28]. These molecules have the advantage of being selectively metabolized by pathways expressed only in bacteria. Further, it is also possible to identify pathways present only in a specific class/species of microorganisms. Metabolized substrates presented the advantage of amplification via enzymatic turnover and specific retention in the cell wall or within other macromolecules resulting in significant accumulation within bacteria as compared to the background host tissues. Several molecules such as [^11^C]-para-aminobenzoic acid (PABA) and 2-[^18^F]-PABA, which target the bacterial folate pathway [33–35]; [^18^F]-labeled maltohexaose and 6-[^18^F]-fluoromaltotriose, specifically transported via the maltodextrin transporter in bacteria [36, 37] derivatives of d-amino acids that are incorporated into the bacterial cell wall [38]; DOTA-Biotin derivatives [39]; and 2-[^18^F]-fluorodeoxysorbitol ([^18^F]-FDS) for Gram-negative Enterobacteriaceae [40] have shown uptake across different strains, maintained selectivity for infection over sterile inflammation in murine infection models. In addition, they can eventually provide information about the causative bacterial class/species and open the possibility of in vivo phenotypization of microorganisms, including the specific antimicrobial susceptibility or resistance, thus resulting in decreasing the inappropriate use of antimicrobials that contributes to the rise of multi-drug-resistant bacteria.

However, the direct visualization of bacteria to diagnose infection requires a 100 to 2000 times higher selectivity of the agent for bacterial accumulation than the background signal [41]. Further, to achieve an adequate sensitivity, a sufficient number of live, replicating microorganisms at the infection site is necessary, to guarantee a signal that is within the spatial resolution of the current clinical imaging system. Therefore, despite excellent pre-clinical studies, radiopharmaceuticals for imaging bacteria in humans are still under development in exciting pre-clinical research.

One of the most widely used strategies in clinical practice is targeting the cell components of the inflammatory host response to infection, such as leukocytes, lymphocytes, or macrophages. This can be achieved by indirect cell targeting using antibodies against NCA-90 (Leukoscan) [42] and NCA-95 (Scintimun) [43] or via the direct radiolabeling of cell subpopulations, such as neutrophils.

In the direct radiolabeling, WBC can be radiolabeled either with ^99m^Tc-hexamethylpropyleneamine oxime ([^99m^Tc]HMPAO, 370–555 MBq) or with [^111^In]oxine (10–18.5 MBq), as detailed in the specific guidelines from the European Association of Nuclear Medicine (EANM) [44] and the Society of Nuclear Medicine and Molecular Imaging [45, 46]. [^99m^Tc]HMPAO radiolabeling is preferred since it allows to perform high-quality SPECT/CT images which are fundamental for the imaging reading also at 24 h, if needed. The standard procedures for patient’s preparation are followed. It is important to be aware if patient is under antibiotic treatment and consider its’ possible effect on WBC uptake when reading the images, but there is no evidence for discontinuation of treatment before the imaging session.

## WBC SPECT/CT

### Patient Preparation

**Fig. 2:**
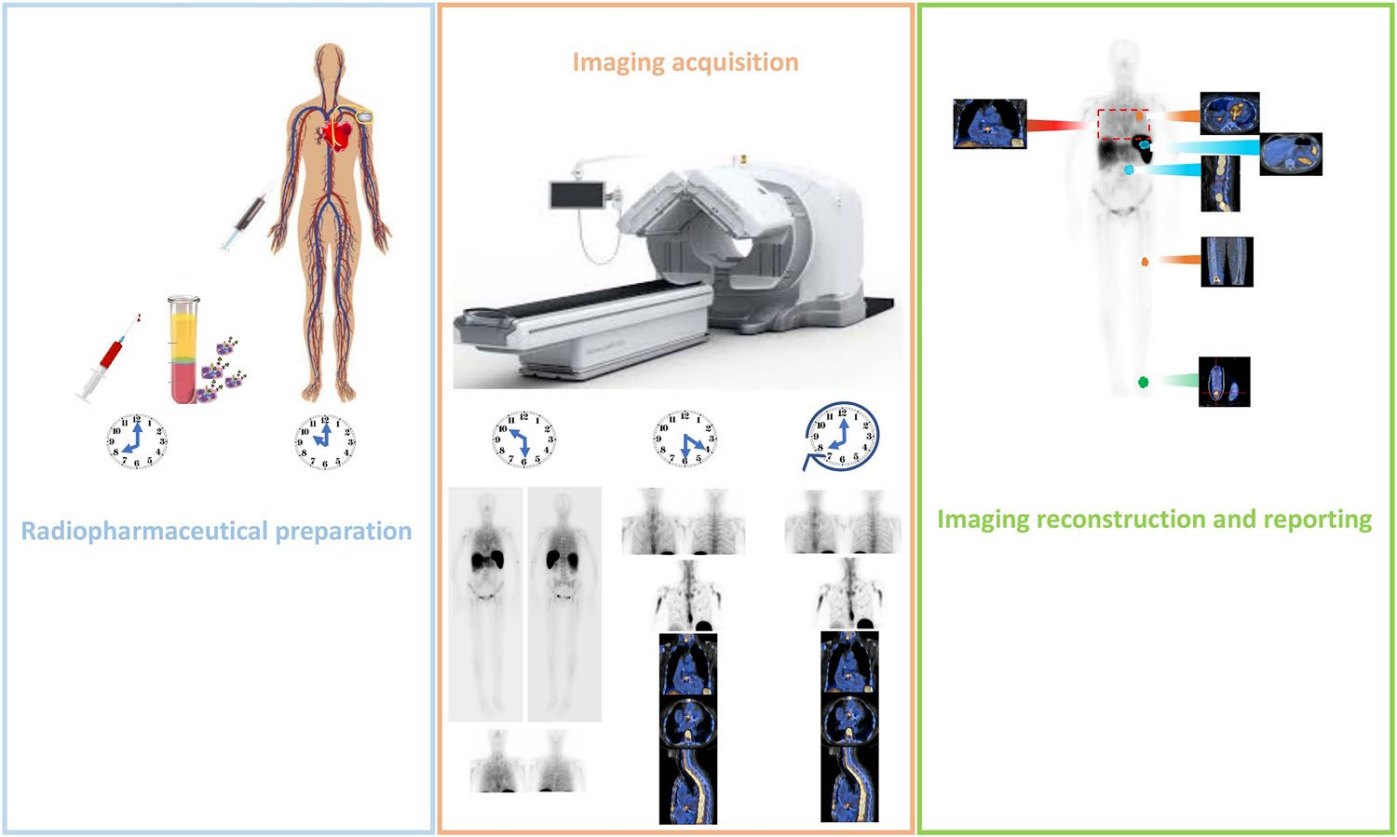
Schematic representation of the flow in radiolabeled WBC scintigraphy. First in the **left panel** (*azul*), the radiopharmaceutical preparation starting with blood sampling and the WBC isolation. After the administration of the radiolabeled cells, the patient is scanned at different time points (**middle panel**, *orange*). Early images (30 min) consists of total-body and spot of the thorax. Late images (4–6 h) and delayed images (20 h) includes spot images and SPECT/CT of the thorax, eventually followed by additional SPECT/CT based on the specific clinical condition. Finally, the images are reconstructed, reoriented, and assessed for the presence of uptake at valve/devices and extracardiac disease involvement as in case of septic embolisms, metastatic sites of infection, and the portal of entry or alternative source of infections (**right panel**, *green*)

In patients with IE or other cardiovascular infections, the standard WBC imaging procedure in terms of patient preparation and WBC radiolabeling preparation is applied [47]. Figure 2 schematically describes the procedure. Generally, the case is discussed within the multidisciplinary Endocarditis Team, as currently recommended by both ESC and AHA guidelines [20••, 48]. A very crucial aspect of WBC imaging in IE and CIED infection is the image acquisition protocol. This should include planar images at 30 min (early), 4–6 h (delayed), and 20–24 h (late) after the reinjection of [^99m^Tc]HMPAO/^111^In-oxine WBC. Early images should include whole body images in addition to chest planar images. SPECT/CT is mandatory to properly identify the site and the extension of infection [23••, 24••]. Specifically, in cardiovascular infections, delayed and late SPECT/CT images are required to increase the diagnostic accuracy of the technique [23••], in addition to confirm and localize findings consistent with infection visualized at planar images (i.e., signal kinetics between 4–6 h and 20–24 h acquisitions stable or increased in uptake intensity or size over time).

The interpretation of WBC scintigraphy begins with a visual quality control of images to check (i) absence of high blood pool activity (suggesting the labeling of a substantial amount of erythrocytes) hampering interpretation even on delayed and late acquisitions; (ii) liver uptake superior to spleen uptake; or (iii) persistent pulmonary uptake, both suggestive of WBC damage prior to reinjection.

Then, SPECT/CT images are visually inspected to diagnose sites of increased WBC uptake, taking into consideration the pattern (focal, linear, diffuse), the intensity, and the relationship to areas of physiologic distribution. Multiplanar reformations (MPR) of the cardiac SPECT/CT images are necessary for proper valve assessment. In case of aortic valve prothesis, images should be reviewed and reconstructed in three different views: left sagittal oblique, left coronal oblique, and cross-sectional oblique views of the valve. For mitral valve prosthesis imaging, reconstruction of 4-chamber, 3-chamber, and 2-chamber views as well as short-axis views of the mitral valve is recommended.

Both CT attenuation corrected and non-corrected SPECT images have to be evaluated in the coronal, transaxial, and sagittal planes, as well as in tridimensional maximum intensity projection (MIP) cine mode. Misalignment between emission and transmission data may generate erroneous correction and thus data misinterpretation. Careful attention should be paid to quality control to avoid reconstruction artifacts. Non-corrected SPECT images become significantly important in the presence of PVs, generators, and electro-catheters due to possible overcorrection artifacts on SPECT/CT images.

Abnormalities detected on WBC imaging should be localized as precisely as possible since at SPECT/CT images, (i) their co-localization with a structural abnormality considered as doubtful on echocardiography will support the hypothesis of infection and (ii) the localization and extent of the disease, on prosthetic material particularly, may contribute to guide surgical procedure.

**Fig. 3:**
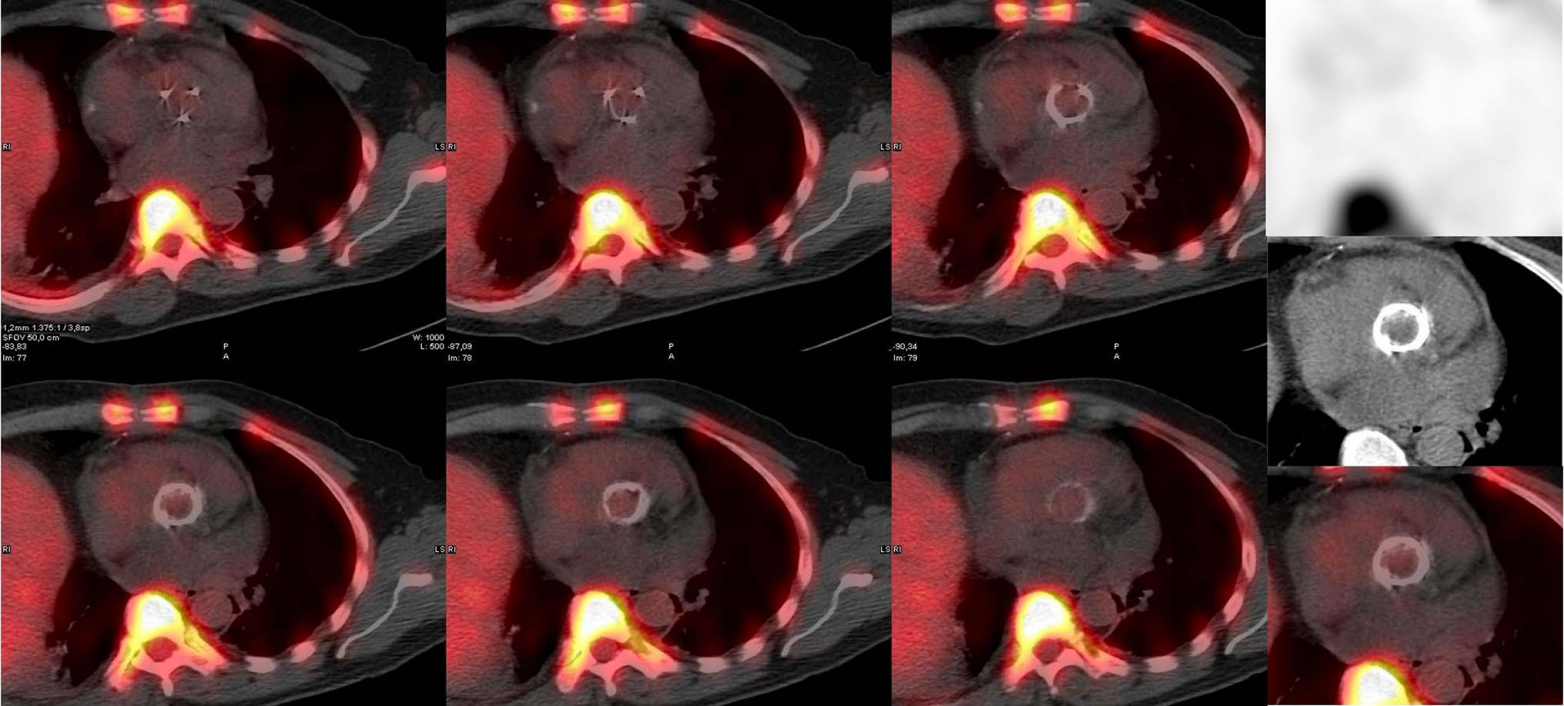
Example of a normal pattern of WBC uptake (^99m^Tc-HMPAO WBC) in a non-infected aortic valve as shown by the re-oriented SPECT/CT images acquired 24 h after the radiopharmaceutical injection (**left panel** superimposed SPET/CT and **right panel** top-down emission, CT, and superimposed SPECT/CT respectively) on Discovery-670, GE Healthcare

**Fig. 4:**
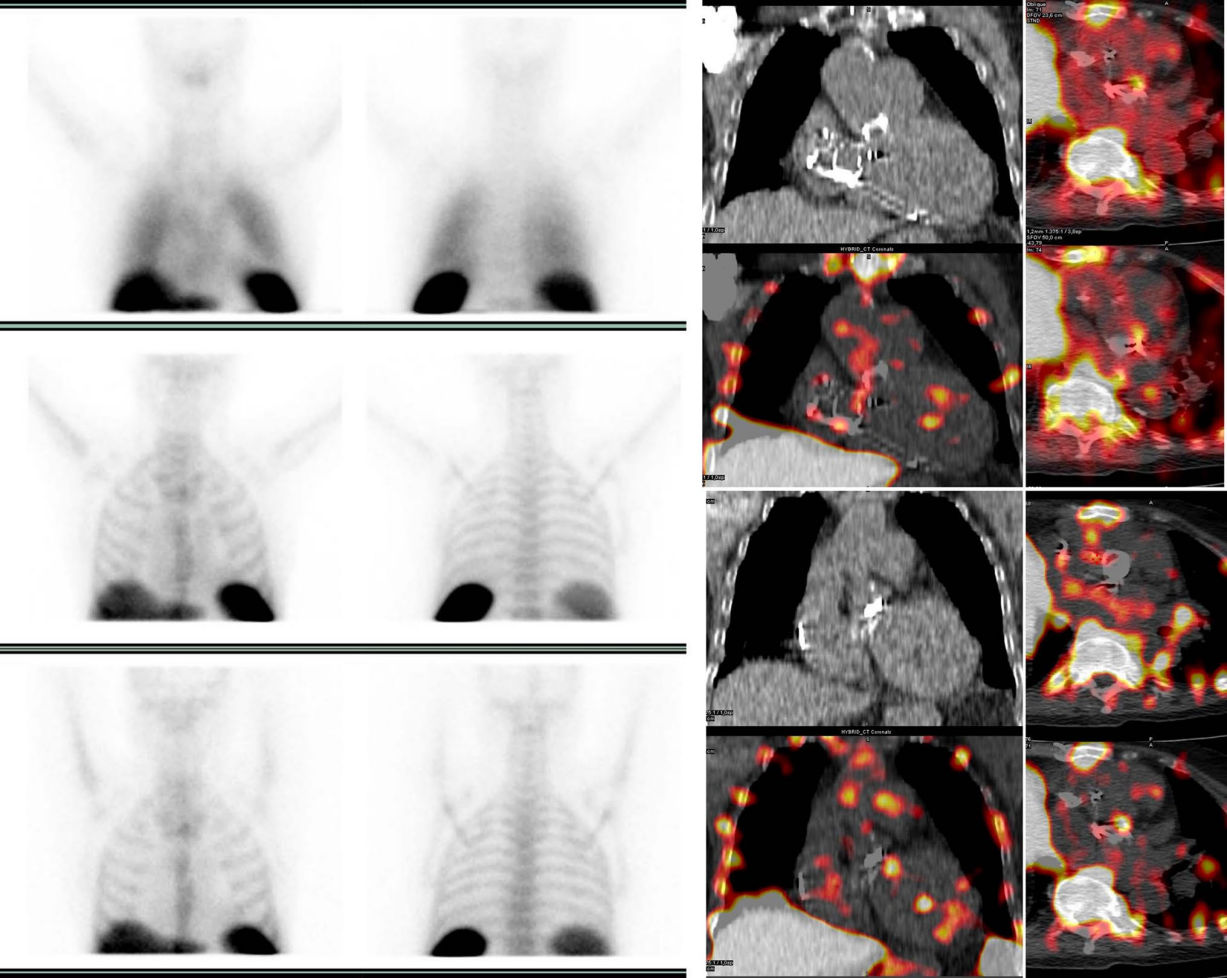
Radiolabeled leukocyte (^99m^Tc-HMPAO WBC) in a patient with suspected IE bearing aortic and mitral prosthetic valve and CIED. Planar images (**right panel** top-down at 30 min, 6 h, and 20 h) after the injection of the radiopharmaceutical and SPECT/CT (**middle column** coronal CT and superimposed SPECT/CT, **left column** transaxial superimposed SPECT/CT at different levels) showing pathological uptake on the lateral and medial sides of the mitral valve prosthesis (images were acquired on Discovery-670, GE Healthcare). No signs of infection are present along the CIED

**Fig. 5:**
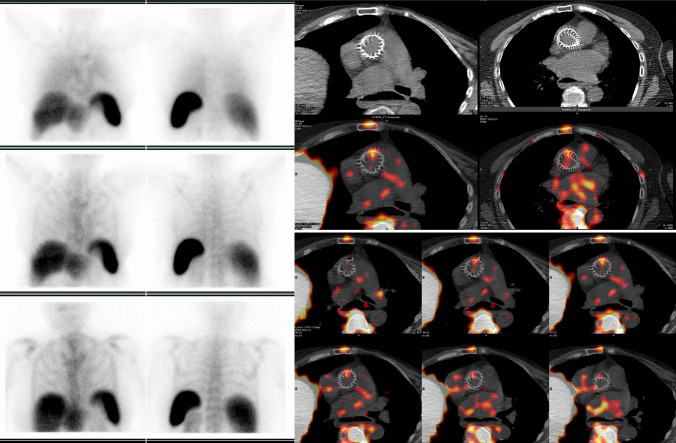
Radiolabeled leukocyte scintigraphy (^99m^Tc-HMPAO WBC) in a patient with suspected prosthetic aortic valve IE. Planar images (**right panel** top-down at 30 min, 6 h, and 20 h) after the injection of the radiopharmaceutical and SPECT/CT (**upper panel**, transaxial CT and superimposed SPECT/CT, **lower panel** transaxial superimposed SPECT/CT at different levels) showing selective uptake on the anterior aspect of the aortic valve prosthesis (images were acquired on Discovery-670, GE Healthcare)

Figure 3 represent an example of normal WBC SPECT/CT imaging in patients with cardiac prosthetic valve while Figs. 4 and 5 represent two clinical examples of WBC SPECT/CT imaging in patients with PVE.

When reading WBC imaging, especially in cardiovascular infections, some important issues should be taken into consideration. Rarely, false positive findings have been described for WBC imaging in IE and CIED infections, even in case of very early infections (Fig. 3, [49••, 50]). On the other hand, false negative scans have been observed in the presence of IE caused by some specific strains [23••].

Following the assessment of the cardiac region, whole body images should be always carefully assessed to search for embolisms as well as for alternative diagnosis and possible sites of uptake which might represent the portal of entry (POE) of the infection (Fig. 2C). Embolic localization may appear at WBC imaging as area of increased uptake over time when located in the brain, lung, and soft tissue. However, when emboli localizations are affecting organs with intense background activity as for normal biodistribution such as the spleen and the bone marrow, they can appear as cold spot (Fig. 7). Since vertebral cold spot might be present in other benign or malignant conditions, such as in the case of vertebral crush or metastasis they are non-specific for infectious embolisms and requires diagnostic confirmation by additional imaging tests. Due to the limited spatial resolution, reduced sensitivity has been described in case of small embolism [51].

**Fig. 6:**
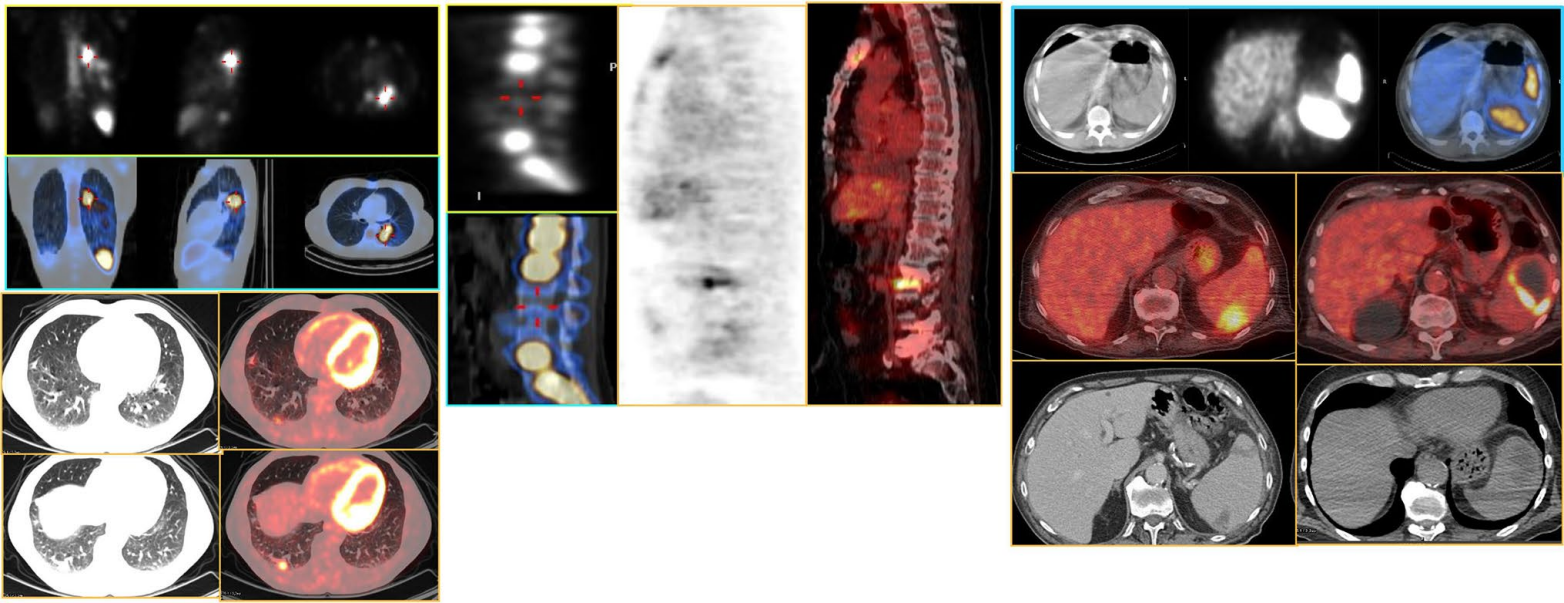
Comparison of WBC and [^18^F]FDG pattern of uptake in embolisms localized in the lung in a patient with right-sided IE appearing. In both cases, there is an area of increase radiopharmaceutical uptake (**right panel** WBC SPECT/CT in the **upper panel** in the emission and superimposed coronal, sagittal, and transaxial views, respectively, and in the **lower panel** [^18^F]FDG PET/CT in the transaxial CT and superimposed images at different levels). In the spine, a different pattern is seen at WBC imaging where typical cold spot is evident due to the normal high uptake of radiolabeled WBC in the normal bone marrow. On the contrary, [^18^F]FDG PET/CT finding shows increased uptake with linear pattern involving the intravertebral space, the lower aspect of the upper vertebral body, and the upper aspect of the lower vertebral body (**middle panel**, from right to left coronal view of WBC upper panel emission and lower panel superimposed SPECT/CT; emission and superimposed coronal [^18^F]FDG PET/CT). Embolism at spleen also presents a different uptake pattern in WBC images where photopenic area of triangular shape is often evident and [^18^F]FDG PET/CT, which can present either uptake or large cold spot (abscess) with a rim of increased uptake (**left panel** from top-down transaxial CT, emission and superimposed SPECT/CT of WBC, superimposed [^18^F]FDG PET/CT and CT)

### Role of WBC Imaging in the Main Clinical Scenarios

#### Left-Sided IE

Sensitivity of WBC SPECT/CT has been reported overall 64–90% with 36–100% specificity, and 85–100% positive and 47–81% negative predictive values [23••, 52•]. In case of abscess formation, WBC SPECT/CT presented 83–100% sensitivity, 78–87% specificity, and 43–71% positive and 93–100% negative predictive values [53], even in the early post-intervention phase [23••, 53]. Figures 4 and 5 represent two examples.

The first large cohort of patients with IE assessed by ^99m^Tc-HMPAO-SPECT/CT was reported by our group in 2012. We described results of ^99m^Tc-HMPAO-SPECT/CT in 131 patients imaged between October 2005 and December 2010. ^99m^Tc-HMPAO-WBC SPECT/CT was true positive and false negative for IE diagnosis in 46/51 and 5/51 of cases, respectively. False negative results were observed in patients receiving high-dose antimicrobial therapy at the time of scintigraphy presenting small valve vegetations (< 6 mm), and infections sustained by *Enterococcus* or *Candida*. We did not observe false positive scans. Septic embolism was detected in 41% of patients. Three cases interpreted as septic embolism at ^99m^Tc-HMPAOWBC scintigraphy were instead false positive because of active vasculitis of the aortic arch, an isolated vertebral metastasis from prostate cancer, and an osteoporotic vertebral crush. Eight scans resulted false negative for extracardiac infection due to kidney or cerebral septic embolism, all detected by CT or MRI [23••].

These data have been later confirmed in a more recent series in which ^99m^Tc-HMPAO-SPECT/CT yields significantly higher diagnostic accuracy, specificity, and PPV than TTE. It helps to differentiate IE infectious and sterile echocardiographic lesions and reduces by 27% the number of misdiagnosed IE classified in the “possible IE” category by modified Duke criteria [54].

Simultaneous ^111^In-WBC/^99m^Tc perfusion imaging was performed using a dedicated cardiac CZT camera in 34 patients with suspected infection of native valves or implants and compared to standard ^111^In-WBC planar scintigraphy and SPECT/CT. Image quality was found superior for CZT imaging vs. conventional SPECT/CT and planar scintigraphy (*P* < 0.01) improving reader confidence for detection of inflammatory foci [52•].

WBC SPECT/CT has an excellent positive predictive value for the detection of IE complications such as perivalvular infection and abscesses in case of PVE. In addition, the intensity of WBC accumulation in the perivalvular area represents a marker of local infectious activity: patients with a mild activity on a baseline scan disappearing on a follow-up scan seem to have a favorable outcome [53]. This open the very interesting perspective of the use of molecular multimodality imaging for the assessment of antimicrobial treatment response.

Rouzet et al. compared the respective performance of WBC scan and [^18^F]FDG PET for the diagnosis of PVE in 39 patients within 14 days. In this study, WBC sensitivity, specificity, positive predictive value, negative predictive value, and accuracy were 64%, 100%, 100%, 81%, and 86%, while [^18^F]FDG PET/CT sensitivity, specificity, positive predictive value, negative predictive value, and accuracy were 93%, 71%, 68%, 94%, and 80%. Discrepant results occurred in 12 patients (31%). True positive PET/CT and false negative WBC occurred in cases of non-pyogenic microorganism IE (*Coxiella* or *Candida*), and false positive PET/CT and true negative WBC scans were all imaged in the first 2 months after the last cardiac surgery [52•].

The most recent hybrid equipment allows to perform WBC SPECT/CTA scan. However, this potential further development has not been yet evaluated.

#### Right-Sided IE

WBC SPECT/CT might be used in all the cases when right-sided IE (particularly PVE) is suspected. Ventilation-perfusion scintigraphy may be an alternative to CT in order to screen septic pulmonary embolism [55]. WBC SPECT/CT and [^18^F]FDG PET/CT(A) have largely substituted ventilation-perfusion scintigraphy allowing the contemporary assessment of right and left side valves, and sites of distant embolisms and POE [25•].

#### Embolic Burden

Extracardiac manifestations in IE (both NVE and PVE) are reported in 30 to 80% of patients. Most frequent are embolic stroke or septic embolization to bone, spleen, or kidneys (84), although only some of these are symptomatic [3, 56]. The majority of embolisms take place within the first 14 days after treatment initiation [57] and they might appear as the initial symptom leading to the diagnosis, and frequently are recurrent [57].

The search for asymptomatic embolic events through systematic extracardiac imaging has become a very important topic, due to the fact that their detection is now considered a minor Duke criterion in the 2015 ESC criteria [20••].

In this setting, a noticeable advantage of WBC SPECT/CT is the possibility to perform the extracardiac work-up within a single imaging procedure, to reveal the concomitant presence of extracardiac infection sites as the consequence of both septic embolism and primary infective processes. However, it should be remembered that in case of embolic events affecting the spleen and the spine, which are characterized by high background activity due to the physiological biodistribution of WBC, embolism might appear as photopenic area, thus requiring further imaging for their final confirmation (Fig. 6).

#### CIED Infections

The objective of the clinical workout in CIED infections is the differential diagnosis among superficial incisional infection, infection involving the skin/subcutaneous tissue versus pocket infection. A further distinction between an infection limited to the generator pocket and infections that extend to the lead and/or CIED systemic infections with eventually IE is also possible. Such differentiation is critical since it is the base to decide the proper adaptive treatment.

The status of the pocket at inspection is extremely important to guide the subsequent imaging management. Indeed, the presence of a clinically positive pocket indicates echocardiograph, which often remains the only imaging tests needed. In all suspected CIED infection, even if only pocket infection is suspected, blood cultures and echocardiographic should be performed. When negative in patients without evidence of pocket infection, no additional imaging is needed. However, in patients with positive blood cultures and negative echocardiographic WBC imaging and PET have proved significant impact for the final diagnosis and they were incorporated in the Novel 2019 International CIED infection criteria [27••]. In case of local infection, the diagnosis is quite straightforward for PET/CT pooled specificity and sensitivity of 93% (95% CI 84–98%) and 98% (95% CI 88–100%), respectively, and AUC of 0.98 at ROC analysis [58•]. The presence of WBC (and [^18^F]FDG) uptake is highly specific for infection (for [^18^F]FDG, there are some limitations very early after implantation), although a negative result does not completely exclude the presence of small vegetations with low metabolic activity (i.e., limited sensitivity and negative predictive value). Therefore, the diagnostic accuracy for lead infections is lower, with overall pooled sensitivity of 65% (95% CI 53–76%), specificity of 88% (95% CI 77–94%), and AUC of 0.861 [58•].

**Fig. 7:**
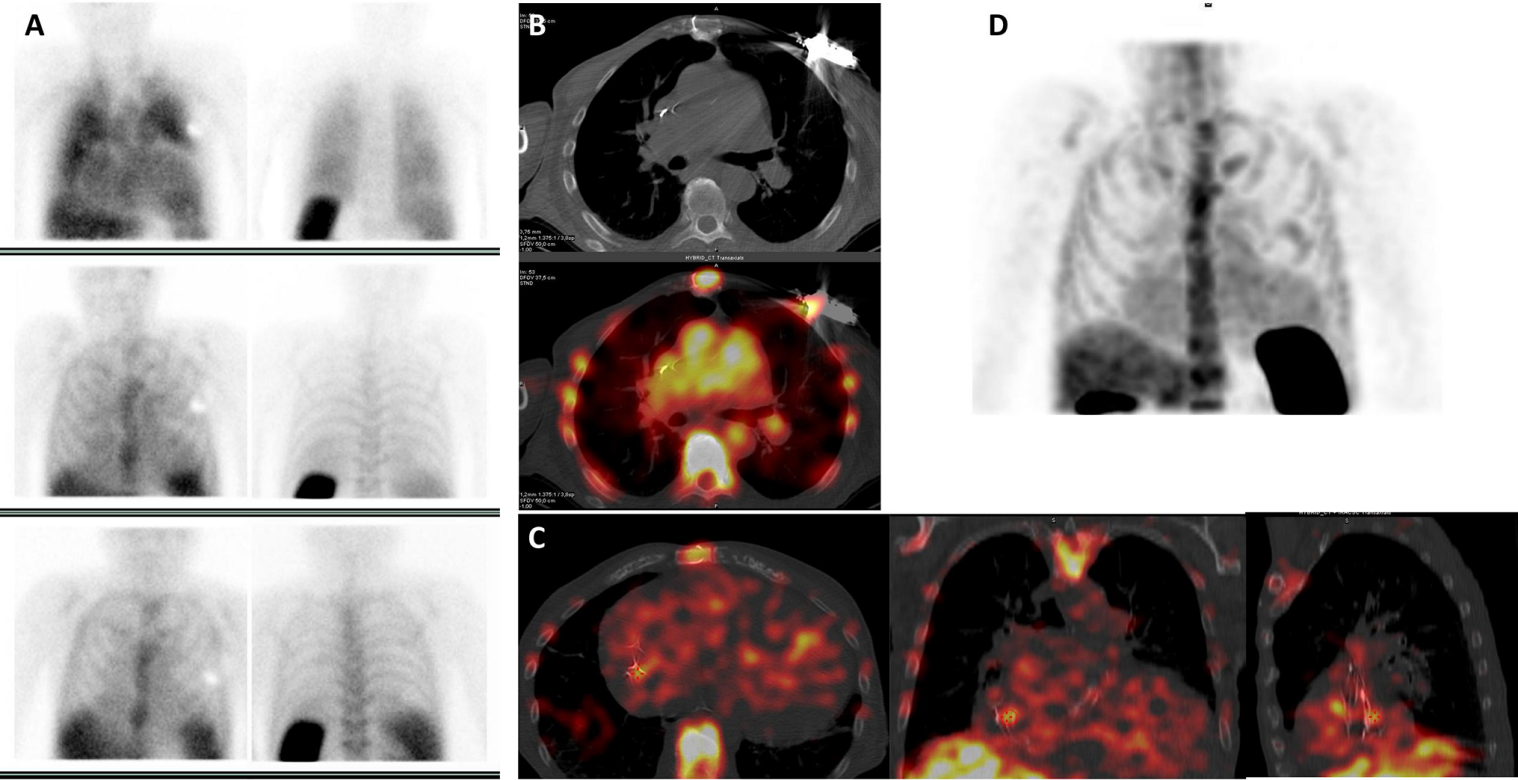
Radiolabeled leukocyte scintigraphy (^99m^Tc-HMPAO WBC) in a patient with suspected CIED infection. Planar images (**A**, top-down at 30 min, 6 h, and 20 h) after the injection of the radiopharmaceutical show an area of pathological leukocyte accumulation that at SPECT/CT (**B**, transaxial CT and superimposed SPECT/CT and **D**, MIP images) is localized at the level of the ICD pocket in the deep posterior portion. In addition, an area of radiopharmaceutical uptake is also evident at the level of the electro-catheter in the right intravascular and interatrial tract (**C**, superimposed SPECT/CT in transaxial, coronal, and sagittal views, respectively)

In the largest study on WBC scan in CIED infections which included 63 patients, sensitivity of 94% and a specificity of 100% were reported [49••]. The main advantage of WBC imaging in this setting was the possibility to differentiate between superficial and deep pocket infection, thus guiding proper patients’ management by medical treatment versus removal of the generator. Further by adding WBC imaging findings with the Duke criteria, it was possible to reclassify most of cases with a “possible” diagnosis, distinguishing infection limited to the pocket or leads from a more severe infection affecting the whole device [49••] and identifying patients requiring device extraction [59]. Figure 7 represents an example.

Also in the case of CIED infections, the possibility to accurately assess the whole body through imaging allows the detection of septic embolisms and the identification of potential infection portal of entry, impacting the subsequent therapeutic management and reducing the risk of relapse [60]. Indeed, detecting lung embolisms in patients with CIED infection, which is a major criterion of the Duke score, has shown to increase the diagnostic sensitivity [61].

#### Composite Aortic Graft

**Fig. 8:**
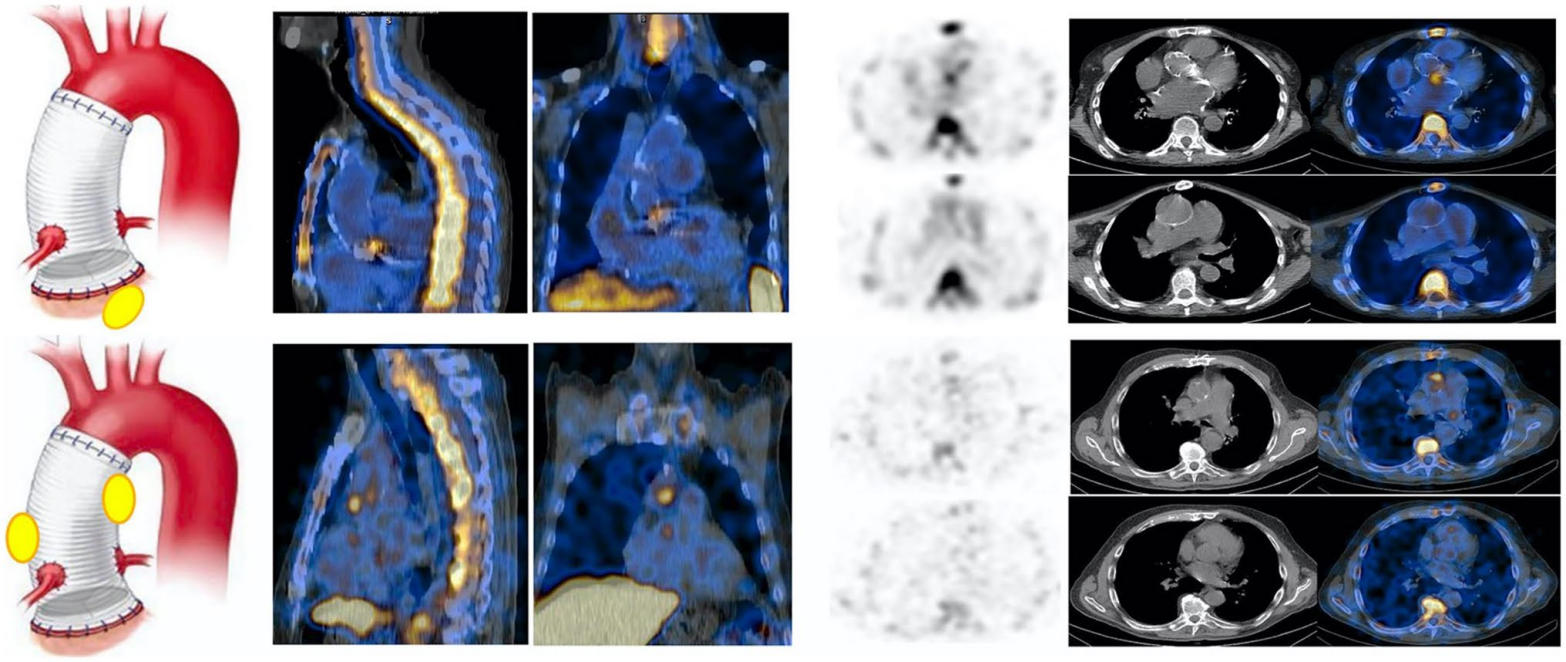
Example of WBC scan in patients with composite aortic valve-ascending aorta prosthesis. At WBC SPECT/CT is possible to differentiate infection involving the aortic valve (**upper panel**), the tube graft (**middle panel**) as shown by the SPECT/CT images of the thorax (from left to right, superimposed sagittal and coronal and transaxial emission, CT and superimposed SPECT/CT images at both the TG and the AI levels). The **left panel** of the figure shows a schematic representation of the final diagnostic category. (Modified from Sollini et al. [63])

The scope of diagnostic imaging in case of suspected infection of composite aortic grafts to differentiate the presence of infection affecting aortic valve (AV)-root from infection localized to the vascular aortic graft (TG). These two conditions might also coexist and involve the surrounding structures such as the mediastinal soft tissue and the sternum. Defining the location and extent of infection and the status of surrounding tissue is crucial for the subsequent treatment planning. In particular, proper diagnosis of infection limited to the vascular portion of the thoracic aortic grafts (VPI, about 2%) [62] is critical due to the high mortality associated with replacement of the graft, especially in patients with long-lasting infections or severe co-morbidities. Alternative options such as graft salvage through aggressive debridement and irrigation or non-surgical management with antibiotics alone [63] can be considered. Currently, there is a lack of specific guidelines for the management of aortic valve-root-vascular prosthesis infections, and the standard recommendations for diagnosis of IE and VPI, including echocardiography and contrast-enhanced CT and/or MRI in short interval, are generally used. TEE is key in the assessment of IE, but the numerous artifacts related to the presence of the prosthesis can result in reduced sensitivity. Accordingly, the majority of the published series underline the need of a diagnostic strategy combining TTE, TEE, CT, and PET/CT to define the final diagnosis [64, 65]. A recent work from our group [66•] demonstrated a sensitivity of 86%, specificity of 92%, and accuracy of 88%, with a slightly higher sensitivity for the detection of TGI as compared to isolated AV and combined AVTG when WBC are used. On the contrary, when specific interpretation criteria are used (focal uptake of intensity > surrounding tissue that persists at both AC and NAC images), [^18^F]FDG PET/CT overall sensitivity, specificity, and accuracy were 97%, 73%, and 90%, respectively. In this series, the specificity of PET/CT increased when considering only patients with non-very early/early infection (< 1 month from surgery). Three out of 22 patients with very early infection had negative WBC SPECT/CT and positive [^18^F]FDG PET/CT, supporting that WBC imaging results are not affected by time from surgery, and reinforced the evidence that WBC SPECT/CT should be preferred early after surgery. This result is also supported by the finding that patients with uncomplicated composite aortic graft implantation prospectively included and studied with [^18^F]FDG PET/CT at either 3 weeks or 1 year after procedure showed no significant differences between PET/CT findings at the two time points, warranting caution in interpretation of PET/CT in the first year after implantation [67]. Figure 8 presents an example of a [^18^F]FDG SPECT/CT in a patient with suspected infection of an aortic valve and ascending aorta prosthesis.

When the diagnosis of infection after Bentall procedure is defined, prompt extracardiac workout is necessary, eventually resulting in the identification of both embolic events or concomitant source of infection/inflammation.

## Conclusions

WBC imaging has significant impact on the ability to promptly diagnose IE and CIED infections providing information about the infection burden, and the location and extent of disease. Further, by extending the sight from the sole heart to the whole body, the technique has contributed to the wider recognition of IE as a systemic disease with the major and more vulnerable location of disease at the heart, but extending outside the heart, resulting in recognition of the importance of accurate infection phenotype for the identification of high-risk patients, a major advance toward developing precision medicine for infectious diseases to optimize individualized therapeutic approaches.
